# Auditory sEMG biofeedback for reducing muscle co‐contraction during pedaling

**DOI:** 10.14814/phy2.15288

**Published:** 2022-05-25

**Authors:** Benio Kibushi, Junichi Okada

**Affiliations:** ^1^ Graduate School of Human Development and Environment Kobe University Kobe Japan; ^2^ Faculty of Sport Sciences Waseda University Tokorozawa Saitama Japan

**Keywords:** augmentation, electromyograms, motor learning, sonification

## Abstract

Muscle co‐contraction between the agonist and antagonist muscles often causes low energy efficiency or movement disturbances. Surface electromyography biofeedback (sEMG‐BF) has been used to train muscle activation or relaxation but it is unknown whether sEMG‐BF reduces muscle co‐contraction. We hypothesized that auditory sEMG‐BF improves muscle co‐contraction. Our purpose was to investigate whether auditory sEMG‐BF is effective in improving muscle co‐contraction. Thirteen participants pedaled on a road bike using four different auditory sEMG‐BF conditions. We measured the surface electromyography at the lower limb muscles. The vastus lateralis (VL) and the semitendinosus (ST) activities were individually transformed into different beep sounds. Four feedback conditions were no‐feedback, VL feedback, ST feedback, and both VL and ST feedback. We compared the co‐contraction index (COI) of the knee extensor‐flexor muscles and the hip flexor‐extensor muscles among the conditions. There were no significant differences in COIs among the conditions (*p* = 0.83 for the COI of the knee extensor‐flexor; *p* = 0.32 for the COI of the hip flexor‐extensor). To improve the muscle co‐contraction by sEMG‐BF, it may be necessary to convert muscle activation into a muscle co‐contraction. We concluded that individual sEMG‐BF does not immediately improve muscle co‐contraction during pedaling.

## Graphical abstract

We constructed EMG biofeedback system for reducing muscle co‐contraction during pedaling. EMG‐BF did not improve the muscle co‐contraction during pedaling. Individual EMG‐BF is difficult to immediately improve muscle co‐contraction during pedaling.

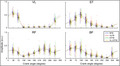

## 1 INTRODUCTION

Muscle co‐contraction between the agonist and antagonist muscles often causes low energy efficiency or movement disturbances (Winter, 2009). High muscle co‐contraction is observed in novice movement. Muscle co‐contraction decreases with motor learning (Thoroughman & Shadmehr, 1999). Effective methods for improving muscle co‐contraction would be useful to facilitate motor learning.

Surface electromyography biofeedback (sEMG‐BF) alters muscle activity patterns and improves biomechanical outputs during movement (Woodford & Price, 2007). For example, applying sEMG‐BF in rehabilitation after the knee joint surgery improves the knee joint range of motion (Xie et al., 2021), peak knee extension torque (Draper & Ballard, 1991), and activation levels of knee extensor muscles (Kirnap et al., 2005). Moreover, employing sEMG‐BF during treatment improves walking ability in stroke patients (Aiello et al., 2005; Jonsdottir et al., 2007) and children with cerebral palsy (Colborne et al., 1994; Dursun et al., 2009). Thus, these studies indicate that sEMG‐BF is an effective rehabilitation support system for several diseases. However, its effect on muscle co‐contraction is unknown. A previous study revealed that the biofeedback of tibialis anterior activity during pedaling altered the structure of muscle coordination patterns (Torricelli et al., 2020). This finding suggests that improving the muscle coordination pattern can be achieved by modulating single muscle activity. Although the effectiveness of sEMG‐BF has been shown, it has been reported that sEMG‐BF has no clear effect on muscle relaxation training and tasks (Davis, 1980; Nielsen & Holmes, 1980). This finding implies that improving muscle relaxation patterns with sEMG‐BF is more difficult than the muscle contraction. To improve muscle co‐contraction, both muscle contraction and relaxation need to be adjusted. It is expected that sEMG‐BF improves muscle co‐contraction if sEMG‐BF includes a system that allows the perception of muscle relaxation, but its effectiveness is unknown.

Visual or auditory feedback are mainly used (Woodford & Price, 2007). A previous study revealed that the auditory sEMG‐BF reduced the frontalis muscle activation, but the visual sEMG‐BF did not (Alexander et al., 1975). Additionally, auditory feedback facilitated higher sensory integration than visual feedback (Ronsse et al., 2011). Moreover, motor learning through the auditory feedback achieved a higher motor learning effect (Ronsse et al., 2011) and higher accurate posture control ability (Hasegawa et al., 2020) even after removing auditory feedback, but the visual feedback did not. Thus, the auditory feedback has an advantage in motor learning. Moreover, it has been reported that the auditory reaction time to the stimuli was shorter than visual (Kohfeld, 1971); thus, muscle activity might be more immediately adjusted by auditory sEMG‐BF than visual sEMG‐BF.

No standard has been proposed for the conversion of sEMG to sound. Understandability, enjoyability, and pleasantness were prioritized in sEMG‐BF for smiling (Nakayama et al., 2017), and therefore, the fatigue in auditory sEMG‐BF (Pauletto & Hunt, 2006) could be prevented by including these factors. Peres et al. (2017) demonstrated that specifically modulating the pitch and loudness of biofeedback sound of sEMG resulted in maximum performance in the muscle activation time estimation task. However, Tsubouchi and Suzuki (2010) state that it was difficult to identify the variation in both pitch and loudness simultaneously. In our study, feedback is required from two muscle activities. Although pure sound feedback, such as a beep, may be undesirable in terms of enjoyability and pleasantness, a simple beep is desirable in terms of understandability.

We hypothesized that sEMG‐BF with the auditory feedback improves the muscle co‐contractions. Our purpose was to investigate whether auditory sEMG‐BF is effective in improving the muscle co‐contraction. This study clarifies the effects of auditory sEMG‐BF from the novel perspective of improving the muscle co‐contraction and contributing to the development of more effective sEMG‐BF methods.

## 2 MATERIALS AND METHODS

### 2.1 Participants

Six women and seven men participated in this study (women: age 25 ± 3 years, height 160 ± 5 cm, body weight 53 ± 4 kg; men: age 25 ± 3 years, height 171 ± 5 cm, body weight 69 ± 9 kg, [mean ± standard deviation]). Participants had no muscular or neurological disorders. All participants self‐reported normal hearing. The participants provided written informed consent to participate after receiving a detailed explanation of the purpose, potential benefits, and risks associated with participation. The experimental procedures were conducted in accordance with the Declaration of Helsinki and were approved by the Local Ethics Committee of the Faculty of Sport Sciences at Waseda University (2020–034).

### 2.2 Experimental setup

Pedaling was selected for the experiment because pedaling consists of relatively simple movements and includes a co‐contraction phase between agonist‐antagonist muscles. Moreover, muscle co‐contraction during pedaling is associated with the skill level. It has been reported that the muscle co‐contraction during pedaling is less in cyclists than triathletes (Candotti et al., 2009), and reducing muscle co‐contraction might be necessary to improve pedaling skills. During the pedal was located around the top dead center, cyclists activated knee extensor muscles (Candotti et al., 2009). During the pedal was located around the bottom dead center, cyclists activated knee flexor muscle to help upward pedaling (Candotti et al., 2009). This has been known as the pull‐up action among cyclists (Mornieux et al., 2008). Not only increasing the agonist muscle activation but also reducing the antagonist muscle activities might be required to improve muscle co‐contraction.

Participants used a road bike (Alu Hydro Triple Butted 6000, Bianchi, Treviglio, Italy) attached to a direct‐drive electromagnetically braked bike‐trainer (Drivo, Elite, Fontaniva, Italy). The bike‐trainer was controlled by a personal computer running My E‐Training software (My E‐Training, Elite srl, Fontaniva, Italy), which was tethered to the bike‐trainer via a USB ANT + dongle (USB ANT Stick™, Garmin, Olathe, Kansas, USA). The road bike was equipped with clipless pedals. We adjusted the saddle height while the participant sat on the seat. During adjustment, participants placed the foot on the pedal and locked the knee with the pedal at the bottom of the stroke (Peveler et al., 2005).

The experiments were conducted in the following order: experiment instructions, initial threshold adjustment of the sEMG‐BF system, adaptation to clipless pedals and the sEMG‐BF system, determination of the workload during the measurement, second threshold adjustment of the sEMG‐BF system, and kinematics and sEMG measurements during the task. To adapt to the clipless pedal and the sEMG‐BF system, participants pedaled for 20 min with the sEMG‐BF system (Mornieux et al., 2008). We needed to adjust the threshold because we constricted the sEMG‐BF system so that it beeps when muscle activation exceeds a certain threshold. We selected the beep sounds based on a previous study that found musical sounds, such as pure tone, acceptable for casual athletes during rowing (Dubus, 2012). Furthermore, the purpose of setting a relatively higher threshold was to prevent a constant beep sound throughout the pedaling cycle.

A detailed description of the threshold adjustment is provided in the next section. To determine the workload during the adaptation and measurement, the heart rate was monitored (Polar H10, Polar Electro Oy, Kempele, Finland). During the adaptation, participants selected the cadence and workload that kept their heart rate around 100–120 bpm. Participants’ agonist and antagonist muscle activities were feedbacked by the beep sound during the adaptation. We asked participants to reduce the overlapping of beep sounds as much as possible. A 10‐minute rest period was provided after the adaptation. A workload during the measurement was defined as that participants could keep their heart rate around 150 bpm and 80‐cadence. During determination of the workload, the workload increased by 20 W per minute until participants could no longer maintain 150 bpm. We considered that we should select a relative workload based on the heart rate because both men and women participated. Mean workloads in the measurement task in women and men were 123 ± 26 W and 176 ± 26 W, respectively. A 10‐min rest period was provided after determining the workload during the measurement tasks.

### 2.3 The EMG‐BF system

The sEMG electrodes (FAD‐DEMG1, 4 Assist, Tokyo, Japan) were attached to participants to measure muscle activity. Test muscles for feedback were the vastus lateralis (VL) and the semitendinosus (ST) on the right side. The sEMG data was imported into a personal computer using an analog data acquisition system (cDAQ‐9179, National Instruments), and the sEMG data was monitored by a custom‐made LabVIEW program (National Instruments, Austin, USA). The sEMG data was measured at 1000 Hz and further processed in real‐time by full‐wave rectification and smoothing with the weighted moving average.

The task for threshold adjustment of the sEMG‐BF system was performed prior to the adaptation to the system and measurement tasks because of different workloads between the adaptation and measurement. In determining a threshold, participants pedaled at the workload used for adaptation or measurement tasks for about one minute. VL and ST were rectified and smoothed to determine each maximum VL and ST value. Thresholds were defined as the normalized sEMG amplitude exceeding 5% of the maximum amplitude during pedaling from the amplitude at the rest. This threshold was determined in preliminary experiments. The threshold was gradually changed, and the participants were asked whether the beep sounds corresponded to the muscle activation timing. We determined the minimum threshold at which an appropriate sound could be continuously produced. Beep frequencies for VL and ST were set at 400 and 800 Hz, respectively.

### 2.4 Measurement tasks

During measurement tasks, participants pedaled for 90 s at 80‐cadence with a determined workload. Four feedback conditions during measurement tasks were: no‐feedback (NFB), VL feedback (VLFB), ST feedback (STFB), and both VL and ST feedback (VL‐STFB). In addition to NFB, the need to compare individual sEMG‐BF as in the previous study (Peres et al., 2017) and sEMG‐BF agonist‐antagonist muscles was assumed. In VL‐STFB, we asked participants not to overlap the beep sound.

### 2.5 Measurement and analysis

To measure pedaling kinematics, reflective markers were attached to the right side of the pedal and crank. These position coordinate values were measured by a three‐dimensional motion capture system with four cameras (OptiTrack Flex 3, NaturalPoint, Inc., Corvallis OR, USA) operating at 100 Hz. We defined the upper dead center as a crank angle of zero. One cycle was defined as the upper dead center to the next upper dead center.

In addition to VL and ST, we measured sEMG signals from the rectus femoris (RF) and biceps femoris (BF) long head muscles to investigate the muscle co‐contraction of hip flexor‐extensor. The hip and knee joints interact with each other during pedaling. We speculated that the biofeedback of the knee flexor–extensor would affect the co‐contraction of the hip flexor–extensor. Since RF and BF activate across multiple phases, their feedback sounds become complicated. As a result, RF and BF were excluded from the feedback system. The RF and BF signals were measured at 1000 Hz (MARQ MQ‐8, Kissei‐Com Tech, Nagano, Japan). The sEMG signals were high‐pass filtered (20 Hz) with a zero‐lag fourth‐order Butterworth filter to remove motion artifacts. Thereafter, the sEMG signals were demeaned, digitally rectified, and low‐pass filtered at 15 Hz with a zero‐lag fourth‐order Butterworth filter (Hagio et al., 2015). The low‐pass filtered sEMG signals were time interpolated over one cycle motion to fit a normalized 200‐point time base. Each muscle activity was normalized to the peak activity for each muscle among all conditions (Kibushi et al., 2018). This normalization method was selected based on the Consensus for Experimental Design in Electromyography project (Besomi et al., 2020). Normalized sEMG data were used to estimate the co‐contraction index (COI), which represents the degree of simultaneous activation between agonist and antagonist muscle. The COI of the hip flexor–extensor (RF–BF) and knee extensor–flexor (VL–ST) was assessed using the following formula (Souissi et al., 2017; Winter, 2009):

$$\begin{array}{c} \text{COI}=\frac{2\times \text{Common Area}}{\left(\text{area}\;EMG_{1}+\text{area}\;EMG_{2}\right)}\times 100 \end{array}$$

where $\text{area}\;EMG_{1}$ and $\text{area}\;EMG_{2}$ represent the integral of the sum of agonist and antagonist sEMG data. Common Area represents the common area between agonist and antagonist sEMG data.

### 2.6 Statistical analysis

Data normality was tested with the Shapiro‐Wilk test. After confirming normal data distribution, repeated measurement of ANOVA was applied to determine the difference in COI among the different conditions. The results with a *p* < 0.05 were considered significant. All statistical analyses were performed using JASP version 0.14.1.0 (JASP Team, 2020).

## 3 RESULTS

**FIGURE 1:**
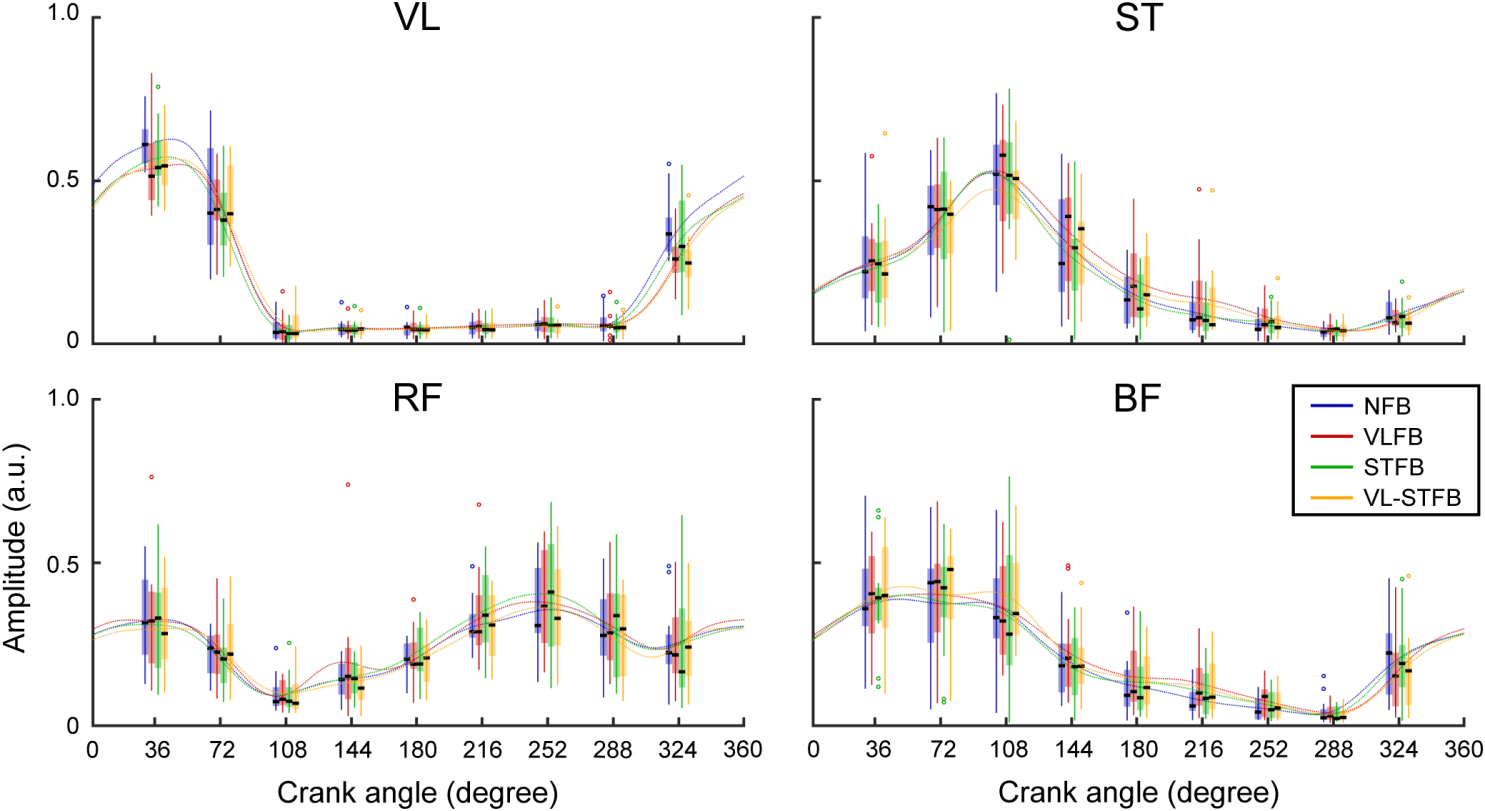
Ensemble‐averaged sEMG among participants. The upper dead center was defined as a crank angle of zero. The upper dead center was defined as a crank angle of zero. Each colored waveform represents the ensemble‐averaged normalized sEMG in a condition among participants. Boxplots on waveforms represent sEMG amplitude in the crank angle of 36, 72, 108, 144, 180, 216, 252, 288, and 324. Circles and horizontal black lines indicate outliers and median, respectively. Upper and bottom edges of lines represent maximum and minimum values, respectively. vastus lateralis (VL), semitendinosus (ST), rectus femoris (RF), biceps femoris (BF), no‐feedback (NFB), VL feedback (VLFB), ST feedback (STFB), and both VL and ST feedback (VL‐STFB)

We qualitatively observed similar activation patterns (peak timing or activation duration) in the ensemble‐averaged sEMG among the conditions (Figure 1). VL mainly activated around the top bottom center. ST activated from 0 to 180 and from 270 to 360° crank angle. RF mainly activated except for 90 to 180° crank angle. BF activated from 0 to 180° crank angle. We observed no significant difference in COIs among the conditions (Table 1).

**TABLE 1:** Average COIs among participants

	Average	SD	*p*‐value	partial $\eta^{2}$
COI of VL‐ST				
NFB (%)	47.8	13.1	0.83	0.02
VLFB (%)	48.8	12.1		
STFB (%)	48.1	13.0		
VL‐STFB (%)	49.2	12.0		
COI of RF‐BF				
NFB (%)	54.1	11.3	0.32	0.09
VLFB (%)	56.5	9.8		
STFB (%)	53.5	12.0		
VL‐STFB (%)	55.9	11.0		

## 4 DISCUSSION

Contrary to our hypothesis, auditory sEMG‐BF does not improve the muscle co‐contractions. This might be because our auditory sEMG‐BF system might have some problems. We suggest an idea for improving muscle co‐contraction by auditory sEMG‐BF.

### 4.1 Improving EMG‐BF to reduce muscle co‐contraction

We considered that controlling muscle relaxation during pedaling is more difficult because modulating force during relaxation is more difficult than force generation (Ohtaka & Fujiwara, 2019). However, inappropriate patterns were observed in both antagonist muscle relaxation and agonist muscle activation. We qualitatively observed that ST activated from 300 to 360° crank angle in this study. This insufficient relaxation of antagonist muscle might cause high COI of VL‐ST around the top dead center. Candotti et al. (2009) observed that the BF activation in triathletes was only active from 0 to 180° crank angle, whereas cyclists were active past the bottom dead center, and they considered that low activation of the BF leads to a higher COI in triathletes. In this study, the ST and BF activation past the bottom dead center were not remarkable in all conditions. This insufficient activation of agonist muscle might cause high COI of VL–ST around the bottom dead center.

The failure to improve muscular co‐contraction in this study could be attributed to an insufficient sEMG‐BF setup. The sEMG‐BF system, specifically, may not be able to fully convert muscle co‐contraction. Peres et al. (2017) reported that modulating the feedback sound of pitch and loudness resulted in the most accurate muscle activation time estimation task (Peres et al., 2017). In this study, constant loudness and different pitches were used so that the participants could easily perceive different muscle activities. When we asked the participants about their perception of muscle activity during the sEMG‐BF system adjustment, they stated that they could perceive each muscle activity as well as the overlap of muscle activities. However, modulating the feedback sound of pitch and loudness may have been more suitable.

In addition to the pitch and loudness issues, the different feedback sounds of sEMG may be because of cognitive overload. In this study, the participants perceived both muscle activation and relaxation in the agonist–antagonist muscles as a single beep or an overlap of different beeps. It has been reported that the simultaneous multiple information of biomechanical output decreases pedaling endurance performance due to information overload (Bayne et al., 2020). Auditory feedback from two muscles could result in multiple information. However, multimodal stimuli are typically perceived more precisely and rapidly than unimodal stimuli (Doyle & Snowden, 2001; Giard & Peronnet, 1999). Sigrist et al. (2013) stated that if workload is high in one modality, augmented feedback should be given in another modality or in a multimodal way. This might prevent cognitive overload and, therefore, might enhance motor learning. Given this perspective, the auditory information of individual muscle activity timing might be insufficient for the modification of muscle co‐contraction. For example, there was no feedback on the severity of muscle co‐contraction in this study. To augment the feedback, visualizing COI might be a solution. Therefore, we might need to convert the signal of individual agonist and antagonist muscle activation into a muscle co‐contraction signal. Furthermore, a system with visual feedback of COI and sound feedback that modulates pitch and loudness based on the muscle co‐contraction signals may be more effective.

Additionally, we might need to modify sound types in sEMG‐BF. Ecological sounds tended to be acceptable for elite athletes during the rowing (Dubus, 2012). Biofeedback with a melodic sound was superior to biofeedback with a rhythmic sound in task acquisition and performance in immediate retention (Dyer et al., 2017). This finding implies that a natural or melodious sound can be acceptable for users and enhance learning. To improve muscle co‐contraction, sEMG‐BF that expresses inappropriate sEMG activation with an ecological or melodious sound might be needed.

### 4.2 Limitations and prospects

There are some limitations to this study. It is unclear whether sEMG‐BF improved the pedal kinetics because the pedal force was not measured. Some studies have reported that biofeedback of the pedal force improves pedal force patterns (O’Brien et al., 2020; Vidal et al., 2020). Therefore, the kinetic characteristics of pedaling might be improved by sEMG‐BF. We should measure the pedal force in a future study. Moreover, the effect of sEMG‐BF in other thresholds or sounds was not investigated. We determined the beep threshold based on the maximum muscle activation values during the pedaling to ensure that the beep sound would be heard while pedaling. Therefore, the threshold in this study is limited during pedaling. We need to investigate beep thresholds based on the maximum voluntary contraction to determine if a common threshold exists to improve muscle co‐contraction among various movements.

We need to investigate whether the sEMG‐BF during unfamiliar or novel movements improves the muscle co‐contraction to reveal movement dependency. Fitts and Posner (1967) divided the motor learning process into three stages: the cognitive stage, associative stage, and the autonomous stage. The participants’ motor learning process in the pedaling motion might be the autonomous stage, where the movement can be achieved unconsciously. At such a stage, the learning process is very slow. To clarify the effects of sEMG‐BF at the autonomous stage, long‐term rather than short‐term interventions might be necessary. Furthermore, effects of sEMG‐BF at different learning processes will become clear by examining the effects of sEMG‐BF at the cognitive stage where rapid learning processes occur and at the associate stage where skills are acquired.

## 5 CONCLUSION

We tested whether sEMG‐BF with beep sounds is effective in improving muscle co‐contraction. We showed that sEMG‐BF did not improve the muscle co‐contraction during pedaling. To improve the muscle co‐contraction by sEMG‐BF, it might be necessary to convert the signal of individual agonist and antagonist muscle activation into a muscle co‐contraction signal. We concluded that individual sEMG‐BF is difficult to immediately improve muscle co‐contraction during pedaling.

## CONFLICTS OF INTEREST

The authors declare no competing financial interests.

## ETHICS STATEMENT

The participants provided written informed consent to participate after receiving a detailed explanation of the purpose, potential benefits, and risks associated with participation. The experimental procedures were conducted in accordance with the Declaration of Helsinki and were approved by the Local Ethics Committee of the Faculty of Sport Sciences at Waseda University (2020–034).

## AUTHOR CONTRIBUTIONS

**Kibushi B:** Conceptualization, Data curation, Formal analysis, Investigation, Methodology, Validation, Roles/Writing ‐ original draft, Writing ‐ review & editing. **Okada J:** Conceptualization, Project administration, Supervision, Validation, Writing ‐ review & editing.
